# Supplementary material for: A high-resolution mRNA expression time course of embryonic development in zebrafish
Source: eLife. 2017 Nov 16;6:e30860. doi: 10.7554/eLife.30860 (PMC5690287; doi:10.7554/eLife.30860)
Supplement: Supplementary file 6. [file elife-30860-supp6.zip › biolayout-clusters-files/Cluster086.html]

Cluster086


# Cluster086: Detail

### Go to ZFA detail

## GO

| GO ID | Description | Domain | Annotated | Expected | Observed | Adjusted p-value | Genes | Ensembl IDs |
| --- | --- | --- | --- | --- | --- | --- | --- | --- |
| GO:0051603 | proteolysis involved in cellular protein... | biological\_process | 208 | 0.34 | 7 | 3.3e-06 | ctslb ctslb si:dkey-239j18.3 zgc:174855 si:dkey-239j18.2 si:dkey-269i1.4 si:dkey-269i1.4 | ENSDARG00000039173 ENSDARG00000074306 ENSDARG00000089806 ENSDARG00000094559 ENSDARG00000095254 ENSDARG00000095744 ENSDARG00000100833 |
| GO:0005615 | extracellular space | cellular\_component | 241 | 0.34 | 8 | 7.1e-07 | ctslb afp4 ctslb si:dkey-239j18.3 zgc:174855 si:dkey-239j18.2 si:dkey-269i1.4 si:dkey-269i1.4 | ENSDARG00000039173 ENSDARG00000041490 ENSDARG00000074306 ENSDARG00000089806 ENSDARG00000094559 ENSDARG00000095254 ENSDARG00000095744 ENSDARG00000100833 |
| GO:0005764 | lysosome | cellular\_component | 64 | 0.09 | 7 | 2.0e-10 | ctslb ctslb si:dkey-239j18.3 zgc:174855 si:dkey-239j18.2 si:dkey-269i1.4 si:dkey-269i1.4 | ENSDARG00000039173 ENSDARG00000074306 ENSDARG00000089806 ENSDARG00000094559 ENSDARG00000095254 ENSDARG00000095744 ENSDARG00000100833 |
| GO:0004197 | cysteine-type endopeptidase activity | molecular\_function | 57 | 0.09 | 7 | 2.4e-10 | ctslb ctslb si:dkey-239j18.3 zgc:174855 si:dkey-239j18.2 si:dkey-269i1.4 si:dkey-269i1.4 | ENSDARG00000039173 ENSDARG00000074306 ENSDARG00000089806 ENSDARG00000094559 ENSDARG00000095254 ENSDARG00000095744 ENSDARG00000100833 |
